# PKC Inhibition by Gö6976 Promotes Osteogenic Differentiation of Dental Follicle Cells Involving Rho-Dependent Pathway Dynamics

**DOI:** 10.3390/biomedicines14081769

**Published:** 2026-08-06

**Authors:** Christian Morsczeck, Anja Reck, Theresa Bodensteiner, Torsten E. Reichert, Hans Christian Beck

**Affiliations:** 1 Department of Cranio- and Maxillofacial Surgery, Hospital of the University of Regensburg, 93053 Regensburg, Germany; anja.reck@ukr.de (A.R.); theresa1.bodensteiner@stud.uni-regensburg.de (T.B.); torsten.reichert@ukr.de (T.E.R.); 2 Department of Clinical Biochemistry, Centre for Clinical Proteomics, Odense University Hospital, 5000 Odense, Denmark; hans.christian.beck@rsyd.dk

**Keywords:** dental follicle cells, osteogenic differentiation, RhoGTPase signaling, Gö6976, sclerostin, RhoA, mineralization

## Abstract

**Background:** Dental follicle cells (DFCs) are promising candidates for regenerative medicine due to their osteogenic potential. While the protein kinase C (PKC) inhibitor Gö6976 is known to enhance DFC differentiation, the underlying molecular mechanisms remain partially understood. **Methods:** A phosphoproteomic analysis of DFCs after 14 days of osteogenic induction was performed. Cells treated with osteogenic differentiation medium (ODM) were compared to those in control medium and ODM supplemented with Gö6976. Reactome pathway analysis identified the RhoGTPase signaling pathway as significantly regulated. This pathway was further validated using PCR arrays, Western blotting, and functional assays (ALP activity, Alizarin Red staining). The impact of RhoGTPase signaling was tested using inhibitors (NSC23766, Y27632, Rhosin) and the activator Geranylgeranyl pyrophosphate (GGPP). **Results:** Phosphoproteomic data highlighted RhoGTPase signaling as a regulatory node. While protein expression of RhoGTPases remained relatively stable, RhoA PCR arrays revealed significant transcriptional regulation after induction of osteogenic differentiation. However, functional inhibition via NSC, Y27632, or Rhosin did not significantly impair basal ODM-induced differentiation; however, Y27632 notably induced SOST expression. Conversely, activation of RhoGTPases via GGPP increased ALP activity and downregulated SOST, suggesting a supportive role of active Rho signaling during differentiation. Crucially, we demonstrated that Gö6976-enhanced mineralization is linked to the activation of RhoA and RhoB. This was confirmed by simvastatin-mediated regulation of Rho expression, which was fully reversed by simultaneous treatment with Gö6976. Furthermore, Rhosin effectively counteracted the pro-osteogenic effects of Gö6976 by inhibiting ALP activity and mineralization while inducing SOST, which is normally suppressed by Gö6976. **Conclusions:** These findings indicate that RhoGTPase signaling, particularly RhoA, is a critical downstream mediator required specifically for the Gö6976-enhanced osteogenic effect in DFCs. We conclude that Gö6976 exerts its stimulatory effect on mineralization by activating RhoGTPases and suppressing the osteogenesis inhibitor SOST, providing a context-dependent mechanism for accelerated differentiation rather than driving basal osteogenesis.

## 1. Introduction

The dental follicle is a dental tissue containing undifferentiated precursor cells for alveolar bone [1,2]. These precursor cells—known as dental follicle cells (DFCs)—can be isolated as colony-forming stem cells and possess a pronounced osteogenic differentiation potential [3,4,5,6]. The osteogenic differentiation of DFCs can be induced, for example, by glucocorticoids such as dexamethasone [5]. In the early phase of osteogenic differentiation, the transcription factor DLX3 has already been identified as a central factor in many osteogenic precursor cells [7,8,9,10]. In DFCs, a positive feedback loop between DLX3 and the Bone Morphogenetic Protein (BMP) signaling pathway was demonstrated for the first time in osteogenic cells; moreover, it is also involved in the induction of the early differentiation marker alkaline phosphatase [11]. Despite these initial findings, the molecular mechanisms in DFCs are extremely complex [12]. One study, for example, has shown that parathyroid hormone-related protein (PTHrP)—a target protein of the Hedgehog signaling pathway—plays a key role in differentiation [13]. In this context, PTHrP fulfills a dual function: while secreted PTHrP regulates alkaline phosphatase activity and DLX3 expression, high endogenous expression correlates with an enhanced differentiation potential [13].

In the later stages of differentiation, regulation by classical protein kinases C (PKCs)—such as PKCα—also gains in importance. We have demonstrated that these kinases inhibit differentiation and that their downregulation influences AKT activity, which, in turn, promotes biomineralization by inhibiting the NF-κB signaling pathway [14]. In parallel, metabolic processes—such as lipid synthesis and the control of oxidative stress—play an essential role in successful differentiation [15,16,17]. Although little is known about the detailed mechanisms by which PKC inhibition supports osteogenic differentiation, we were able to demonstrate in a previous study that the expression of the sclerostin gene decreased following PKC inhibition by GÖ6976, and that the regulation of sclerostin mediates the mineralization of DFCs induced by PKC inhibition [18].

Since the molecular processes that specifically regulate the later stages of osteogenic differentiation remain insufficiently understood, the present study focused specifically on the inhibition of PKC within this time window. To this end, we performed a phosphoproteomics analysis on day 14 of differentiation [19]. Here, we were able to demonstrate that the regulation of apoptosis and oxidative stress plays a crucial role in osteogenic differentiation. In this earlier study, it was also possible to draw conclusions indicating that signaling pathways associated with RhoGTPases play an important role [19]. To further elucidate the role of classical PKCs during this phase, the specific inhibitor GÖ6976 was employed in our new study. The bioinformatic analysis of the resulting data once again identified regulated signaling pathways associated with Rho GTPases as particularly significant. The functional importance of Rho GTPases had previously been demonstrated in an earlier study highlighting the significance of downstream ROCK activation in periodontal ligament (PDL) cells. During the osteogenic stimulation of PDL cells, a massive reorganization of the cytoskeleton occurs through the induction of F-actin stress fibers [20]. If this process is blocked by the ROCK inhibitor Y-27632, the expression of essential ECM genes—such as Fibronectin-1 and Collagen Type I—drops drastically, thereby almost completely reversing the osteogenic phenotype. Moreover, Wang et al. [21] demonstrated that the administration of Y-27632 massively promotes the in vitro expansion and migration of periodontal stem cells via the ERK cascade, while temporarily halting differentiation. In a more recent study utilizing a mechanical stretch model, Ye et al. [22] showed that mechanical tension stimulates bone formation via the RhoA/ROCK signaling axis. As part of this study, we investigated Rho GTPase-mediated processes and evaluated the significance of inhibitors of RhoA (Rhosin) and ROCK (Y-27632) for the osteogenic differentiation of DFCs.

## 2. Materials and Methods

### 2.1. Cell Culture and Osteogenic Differentiation

Human dental follicular cells (DFCs) were obtained from ALL cells (Alameda, CA, USA). No patient-specific clinical data or donor demographics (such as age) were available to the investigators. The standard culture medium (DMEM) was Dulbecco’s Modified Eagle Medium (DMEM; Sigma-Aldrich, St. Louis, MO, USA) supplemented with 10% fetal bovine serum (FBS; Sigma-Aldrich) and 100 µg/mL penicillin/streptomycin. DFCs after passage 6 were used for experiments.

DFCs were cultured until subconfluence (>80%) in standard cell culture medium before osteogenic differentiation was stimulated with osteogenic differentiation medium. The differentiation medium (ODM) was a DMEM-based cell culture medium containing 10% fetal bovine serum (Sigma-Aldrich), 50 μmol/L ascorbic acid-2-phosphate, 10 mmol/L KH_2_PO_4_, HEPES (20 mmol/L), 10 nM dexamethasone and 100 μg/mL penicillin/streptomycin (Sigma Aldrich). The differentiation of DFCs was determined based on the activity of alkaline phosphatase (ALP), which was measured after 7 days of osteogenic differentiation. DFCs were lysed (Triton-X solution) and the lysates were assayed for ALP activity with a solution containing p-nitrophenyl phosphate (60 min at 37 °C). The production of p-nitrophenol was measured spectrophotometrically at 415 nm. The measured values of the ALP test were related to the measured values (490 nm) for the protein concentration determined with the BCA test (Thermo-Fisher, Waltham, MA, USA). ALP activity values were shown as relative unites. Differentiation with respect to the mineralization of the extracellular matrix was also evaluated after 28 days, as detected by Alizarin Red staining. Cells were fixed with 4% formalin and stained with Alizarin Red Solution (Merck Millipore, Billerica, MA, USA). The stains were quantified by dissolving alizarin red crystals in cetylpyridinium chloride and measuring the optical density (595 nm).

For functional inhibition and activation studies of RhoGTPase-associated signaling, DFCs were treated with the following small molecules at the indicated final concentrations: NSC23766 (10 µM), Rhosin (10 µM), Y27632 (10 µM), Simvastatin (125 nM), and GGPP (10 µM). These molecules were added to the osteogenic differentiation medium (ODM).

For the analysis of the phosphoproteome, DFCs were cultured in osteogenic differentiation medium (ODM) as well as in ODM supplemented with the PKC inhibitor GÖ6976 (100 nM). DFCs in standard medium (DMEM) served as a control (Ctrl). Treatment with GÖ6976 was initiated on day 13 of differentiation. Total proteins were isolated exactly 14 days after the induction of osteogenic differentiation.

For protein extraction, DFCs were harvested by trypsin treatment. The cells were lysed with buffer (20 mM Tris, 48 mM NaF, 150 mM NaCl, 2 mM Na orthovanadate, 1% NP-40 and 10% glycerol) with freshly added protease (Roche, Mannheim, Germany) and phosphatase (Sigma) inhibitor cocktails on ice for 30 min.

### 2.2. Western Blot Analysis

The protein concentration of the cell lysates was determined using the BCA (bicinchoninic acid) Protein Assay Kit (Thermo-Fisher) according to the manufacturer’s instructions, with bovine serum albumin (BSA) serving as the standard. For the subsequent analysis of protein expression, equal amounts of protein were electrophoretically separated via SDS-PAGE on Mini-PROTEAN TGX Stain-Free Precast Gels (Bio-Rad, Hercules, CA, USA). Prior to transfer, the gel was activated using UV light in the ChemiDoc MP Imaging System (Bio-Rad) to visualize the proteins via the covalent modification of tryptophan residues. Protein transfer to a PVDF membrane was performed using the Trans-Blot Turbo Transfer System (Bio-Rad). To validate uniform sample loading and transfer efficiency, the total protein pattern of the membrane was documented immediately after transfer using the Stain-Free feature. After blocking non-specific binding sites (1 h in 5% *w*/*v* non-fat dry milk or BSA in TBST), the membranes were incubated overnight at 4 °C with primary antibodies from Cell Signaling Technology (Danvers, MA, USA). The antibodies used included the Rho-GTPase Antibody Sampler Kit (#9968) (for the detection of RhoA, RhoB, RhoC, Rac1/2/3, and Cdc42), specific antibodies against ROCK1 (#4035) and ROCK2 (#9029), and the Rho-specific antibody from the Active Rho Detection Kit (#8820) (each at a dilution of 1:1000). Immunocomplexes were detected via chemiluminescence on the ChemiDoc system following a one-hour incubation with HRP-conjugated secondary antibodies, using the Stain-Free signal of the membrane as a reference for sample loading.

### 2.3. Quantitative Reverse Transcription Polymerase Chain Reaction (qRT-PCR)

Total RNA was isolated from cells with an RNeasy isolation kit (Qiagen, Hilden, Germany). The cDNA synthesis was performed with total RNA and the iScript™ Advanced cDNA Synthesis Kit for qRT-PCR (Bio-Rad) according to the manufacturer’s protocol. PCRs were made with SsoAdvanced™ Universal SYBR^®^ Green Supermix (Biorad) on the StepOne real-time PCR machine (Life Technology, Carlsbad, CA, USA). Primers for the Sclerostin (SOST) gene were purchased from Bio-Rad. For relative gene expression in a single sample, the SOST gene expression was normalized to the gene expression of the RPS18 gene (housekeeper gene) and calibrated to the relative gene expression in a control group using the ΔΔCt method.

Analysis of gene expression within specific signaling pathways was performed using PrimePCR Pathway Arrays (Bio-Rad) for the “RhoA regulation pathway” and the “RhoB regulation pathway”. The associated Bio-Rad CFX Manager software (Version 3.1, Bio-Rad) was utilized to identify significantly regulated genes. Statistical analysis and visualization of differentially expressed genes were presented in the form of volcano plots. Genes were classified as significantly regulated if they met a predefined cutoff (*p*-value < 0.05 and a minimum twofold change in expression/fold change > 2).

### 2.4. Phosphoproteomic Analysis

Precipitated protein extracts were reduced, blocked with iodoacetamide, trypsinised and labeled with the 16-plex tandem mass tag (TMT) reagent according to the manufacturer’s protocol (Thermo Fisher Scientific, Bremen, Germany ). Samples were tagged with mass tags 127N through 134N and mixed with a pool of all samples (mass tag 126). The resulting peptide mixture (ca. 400 µg) were subjected to phosphopeptide enrichment using titanium dioxide (TiO_2_) virtually as previously described (PMID: 28116542). The purified phosphopeptide mixtures were fractionated into seven fractions using high pH fractionation, also as previously described [23], and analyzed by nanoLC-MS/MS as described below.

NanoLC-MS/MS analysis was performed on a Orbitrap Astral mass spectrometer (Thermo Fisher Scientific) (OTOT mode) equipped with a nanoHPLC interface (Dionex UltiMate 3000 nano HPLC). Samples (5 µL) were loaded onto a custom fused capillary guard column (2 cm length, 360 µm OD, 75 µm ID, packed with ReproSil Pur C18 5 µm resin (Dr. Maisch HPLC GmbH, Ammerbuch, Germany) with a flow of 4 µL/min for 7 min. Captured peptides were separated on a custom-designed fused capillary column (20 cm length, 360 µm OD, 75 µm id, packed with ReproSil Pur C18 1.9 µm resin) using a linear gradient from 90–86% buffer A (0.1% formic acid) to 27–32% buffer B (80% acetonitrile in 0.1% formic acid) over 40 min with a flow rate of 250 nL/min. Mass spectra were acquired in positive ion mode, using automatic data-dependent switching between an orbitrap MS scan in the 350–1400 *m*/*z* mass range followed by higher-energy collision dissociation (HCD) fragmentation of the selected peptide followed by orbitrap detection of fragmented peptide ions. The normalized AGC target for MS1 was 200% at 60 kDa resolution, and normalized AGC target for MS2 was 200% at 45 kDa resolution. Isolation window was 0.7 Da. Fragmentation in the HCD cell was performed with normalized collision energies of 35 eV. The ion selection threshold was 20,000 counts, and selected sequenced ions were dynamically excluded for 60 s.

All Astral raw data files were processed and quantified using Proteome Discoverer version 3.0.1.27 (Thermo Fisher Scientific). The Sequest and MS-Amanda search engines built into Proteome Discoverer were used to search the data using the following criteria—Protein Database: Uniprot (downloaded 10 January 2024, 20,330 entries) and restricted to humans. Fixed search parameters included trypsin, allowing a missed cleavage, carbamidomethylation at cysteines, and TMTpro labeling at lysine and N-terminal amines, while serine, threonine, and tyrosine phosphorylation, methionine oxidation, and deamidation were set as dynamic. The mass tolerance of the precursor was adjusted to 8 ppm and the mass tolerance of the fragment was adjusted to 0.05 Da. FDR was calculated using a decoy database search and only peptide identifications with high confidence (misrecognition rate < 1%) were included.

Identified regulated phosphoprotein peptides from different groups were compared and selected according to significance (Student’s *t*-test *p* < 0.05; ratio > 2 for comparison ODM vs. DMEM; ratio > 1.5 for comparison ODM+GÖ6976 vs. ODM) were compared with the analysis databases Reactome (reactome.org) and DAVID Bioinformatics Resources (david.ncifcrf.gov) [24,25].

### 2.5. Statistical Analysis

Data are presented as mean ± standard deviation (SD). For comparisons between two experimental groups, the two-tailed Student’s *t*-test was applied. For multiple comparisons involving more than two groups, a one-way analysis of variance (ANOVA) followed by Tukey’s post hoc test was used to determine statistical significance. Differences were considered statistically significant at *p* < 0.05 and marked with an asterisk (*).

## 3. Results

### 3.1. RhoGTPase Signaling Is a Key Regulator During Osteogenic Differentiation of DFCs

**Figure 1:**
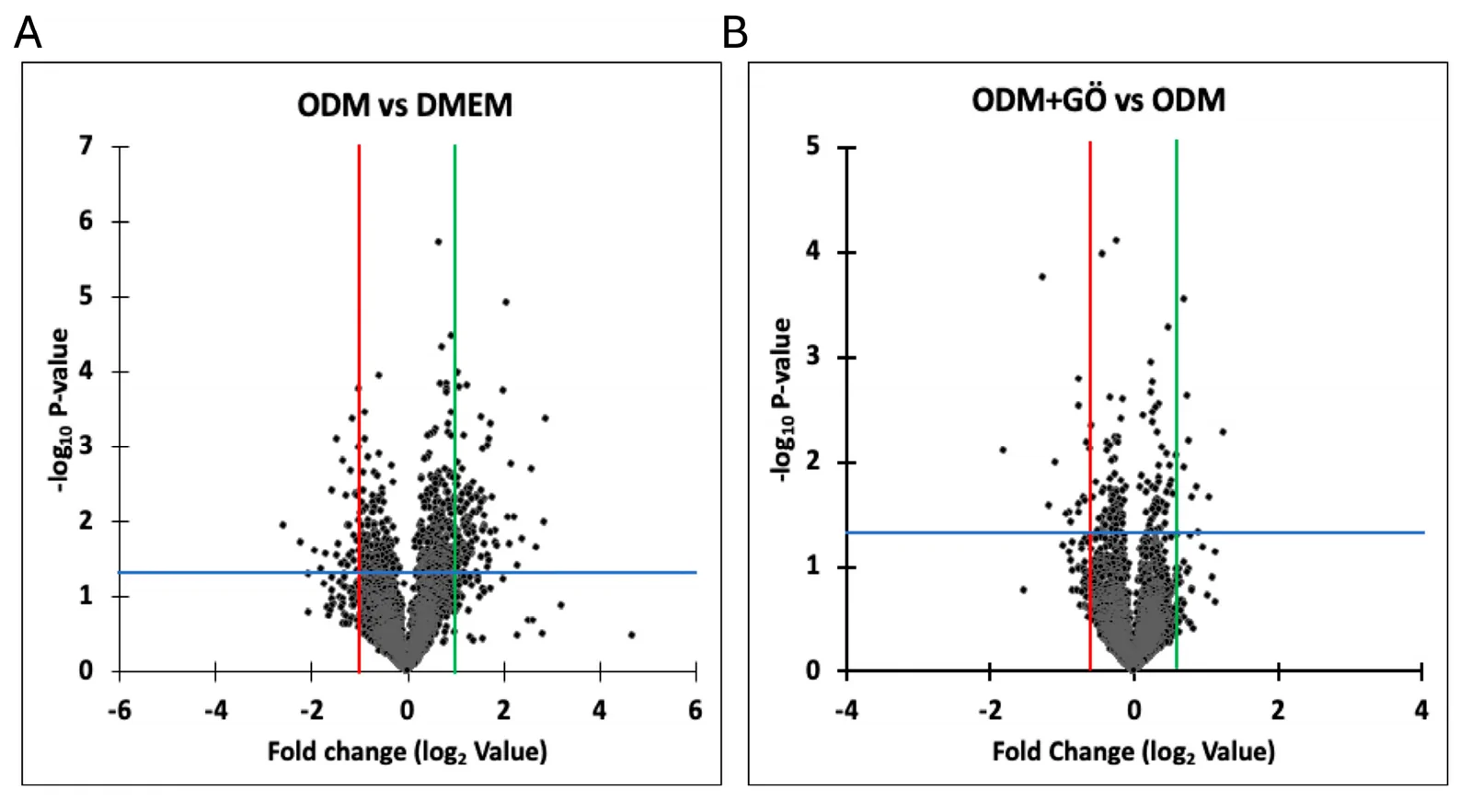
Volcano plots of regulated phosphopeptides. Statistical significance (log_10_ *p*-value) is plotted against the relative abundance change (fold change; log_2_ value) for identified phosphopeptides (n = 3 biological replicates). Horizontal and vertical lines indicate the thresholds for statistical significance (Student’s *t*-test, *p* < 0.05) and fold change, respectively. (**A**) For the comparison of ODM vs. DMEM, significantly regulated peptides were selected using a ratio threshold of >2. (**B**) For the comparison of ODM+GÖ6976 vs. ODM, significantly regulated peptides were filtered using a ratio threshold of >1.5.

To investigate the acute signaling cascades regulated by PKC inhibition during active differentiation, DFCs were first pre-cultured in ODM for 13 days to initiate osteogenesis. On day 13, cells were stimulated with GÖ6976 for an acute window of 24 h prior to harvesting for phosphoproteomic screening (Figure 1) and mechanistic assays. This specific time point was chosen to capture dynamic phosphorylation shifts within an established osteogenic environment, distinct from the continuous, long-term GÖ6976 treatment required to visually assess matrix mineralization via Alizarin Red staining at day 28 (Figure 5A).

Volcano plot analyses were subsequently performed to screen for significantly regulated phosphopeptides across the different experimental groups (Figure 1). Phosphopeptides were selected based on statistical significance (Student’s *t*-test, *p* < 0.05, n = 3) using specific ratio thresholds. For the comparison of ODM vs. DMEM (Figure 1A), peptides were filtered using a standard ratio threshold of >2. However, for the comparison of ODM+GÖ6976 vs. ODM (Figure 1B), applying a strict threshold of >2 yielded only six significantly regulated proteins, a sample size insufficient for robust downstream bioinformatic pathway enrichment. To enable a statistically valid analysis and comprehensively capture the coordinated signaling alterations induced by the kinase inhibitor, the ratio threshold was adjusted to >1.5, which expanded the dataset to 26 regulated proteins.

**Figure 2:**
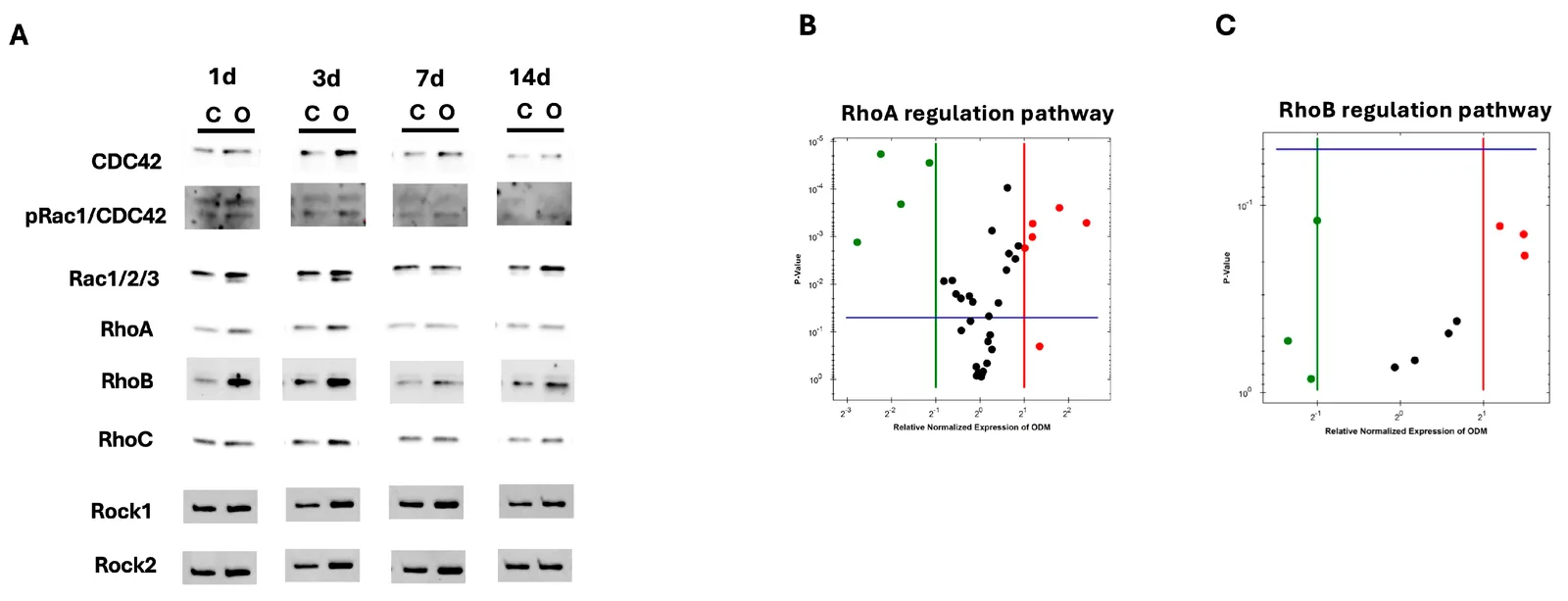
RhoGTPase expression and signaling during osteogenic differentiation. (**A**) Representative Western blot analysis of RhoA, RhoB, RhoC, Rac1/2/3, pRac1/Cdc42, Rock1, and Rock2 in DFCs at days 1, 3, 7 and 14 of differentiation. Lanes are labeled C for Control (DMEM) and O for ODM. Note the relatively stable protein expression across groups. (**B**,**C**) Gene expression profiling using PrimePCR Pathway Arrays for RhoA (**B**) and RhoB (**C**) regulation after 3 days of differentiation. Volcano plots highlight significantly regulated genes (*p* < 0.05, Fold Change > 2) in the RhoA pathway, while the RhoB pathway shows minor transcriptomic shifts.

Comparative analysis of ODM vs. DMEM (Table 1, Table 2, Tables S1 and S3) and ODM vs. ODM + GÖ6976 (100 nM) (Table 3, Table 4, Tables S2 and S4) using Reactome and Gene Ontology (GO) successfully identified the RhoGTPase signaling pathway and proteins associated with cytoskeleton organization as significantly regulated. To further validate these findings, we analyzed the pathway components at different molecular levels. While Western blot analysis showed only weak regulation of total RhoGTPase protein expression during differentiation (Figure 2A), PrimePCR arrays revealed significant transcriptomic changes specifically within the RhoA signaling pathway, whereas RhoB was less affected (Figure 2B).

### 3.2. Activation, but Not Inhibition, of RhoGTPases Modulates Osteogenic Potential

**Figure 3:**
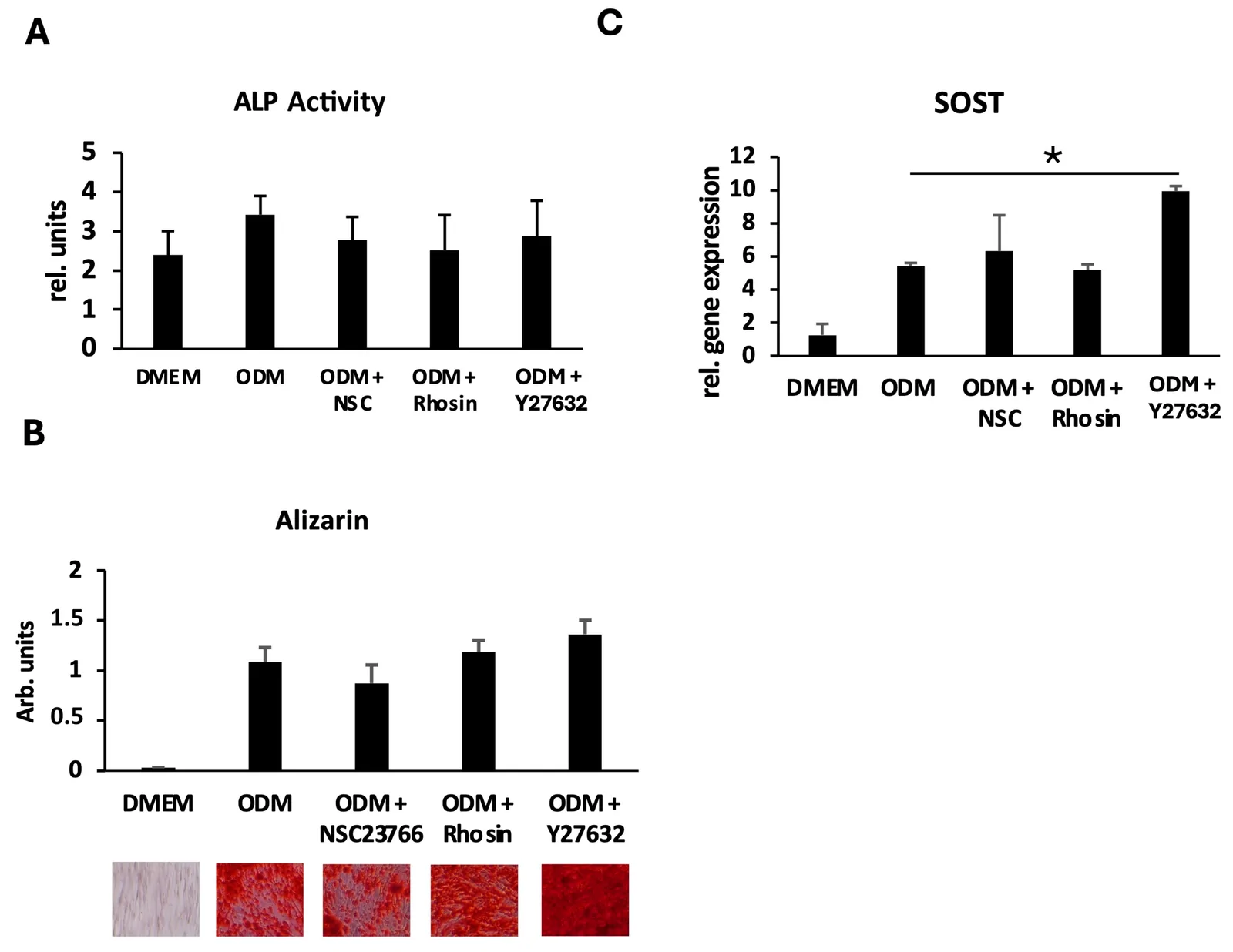
Impact of RhoGTPase inhibition on DFC differentiation. (**A**) ALP activity assay and (**B**) Alizarin Red staining of DFCs treated with inhibitors NSC23766, Y27632, or Rhosin in ODM. No significant reduction in mineralization or ALP activity was observed compared to ODM alone. However, (**C**) Y27632 treatment led to a significant upregulation of *SOST* mRNA expression (*p* < 0.05). Data are presented as mean ± SD (n = 3). Differences were considered statistically significant at *p* < 0.05 and marked with an asterisk (*).

**Figure 4:**
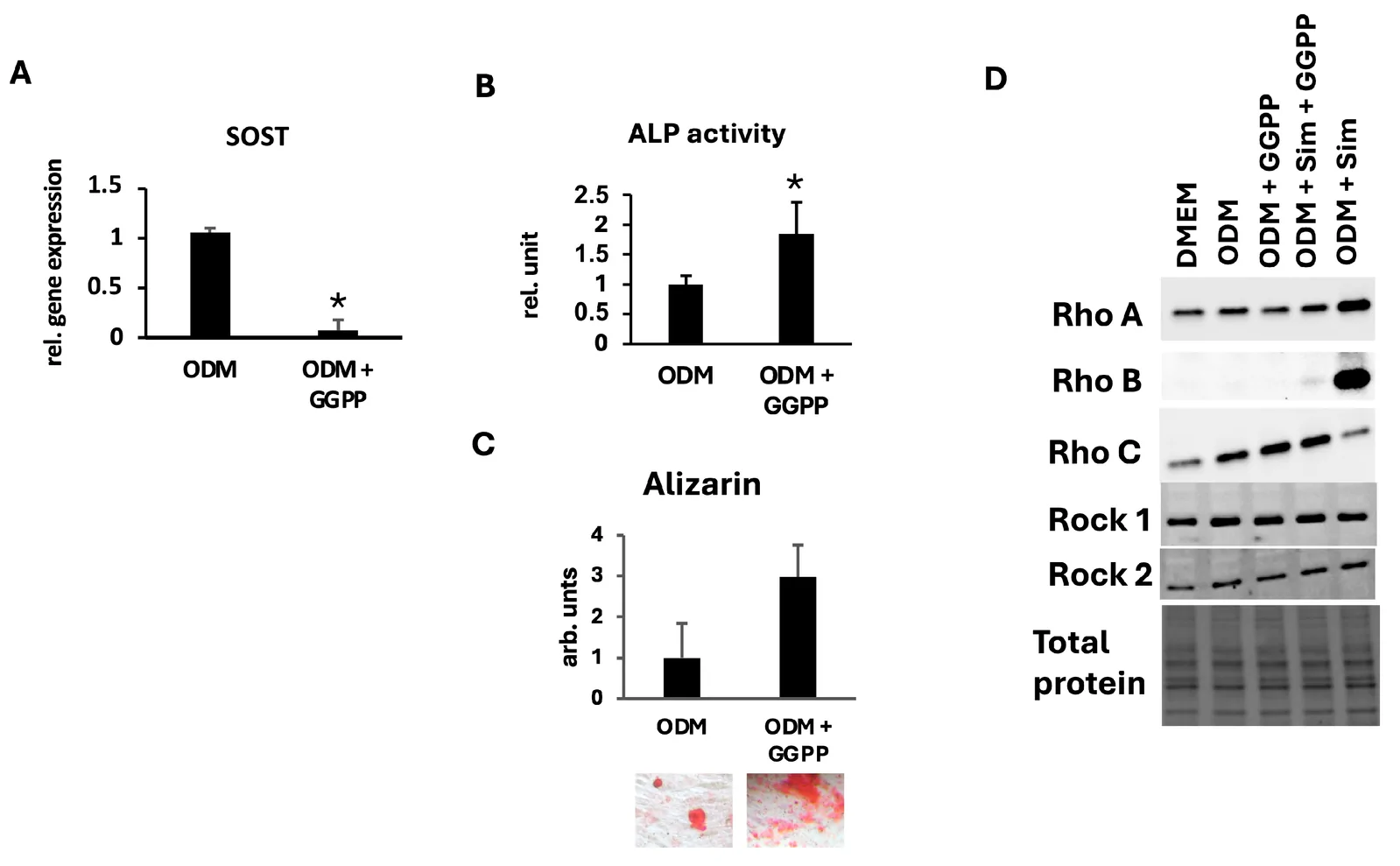
Activation of RhoGTPases by GGPP enhances osteogenic markers. DFCs were treated with GGPP to induce RhoGTPase activation. (**A**) GGPP treatment significantly suppressed *SOST* expression after 3 days of osteogenic differentiation in ODM. (**B**) GGPP increased ALP activity. (**C**) Alizarin Red staining showed a positive trend but did not reach statistical significance. Data are presented as mean ± SD (n = 3). (**D**) Western blot analysis demonstrates that GGPP stabilizes the protein expression levels of RhoA, RhoB, and RhoC against simvastatin-induced fluctuations. Differences were considered statistically significant at *p* < 0.05 and marked with an asterisk (*).

To evaluate functional relevance, DFCs were treated with the inhibitors NSC 23766, Y27632, and Rhosin. Interestingly, these inhibitors did not significantly alter ALP activity or mineralization (Alizarin Red staining) (Figure 3A,B). However, inhibition of ROCK by Y27632 significantly induced the expression of the mineralization inhibitor SOST (Figure 3C). Conversely, activation of RhoGTPases via geranylgeranyl pyrophosphate (GGPP) treatment inhibited SOST expression and significantly increased ALP activity, although Alizarin staining showed only a non-significant upward trend (Figure 4A–C). Furthermore, Western blot analysis demonstrated that GGPP prevented simvastatin-induced changes in RhoA, RhoB, and RhoC protein levels in ODM (Figure 4D). Consequently, these data suggest that Rho GTPase signaling actively modulates osteogenic differentiation.

### 3.3. GÖ6976 Promotes Mineralization by Activating RhoGTPase Signaling

**Figure 5:**
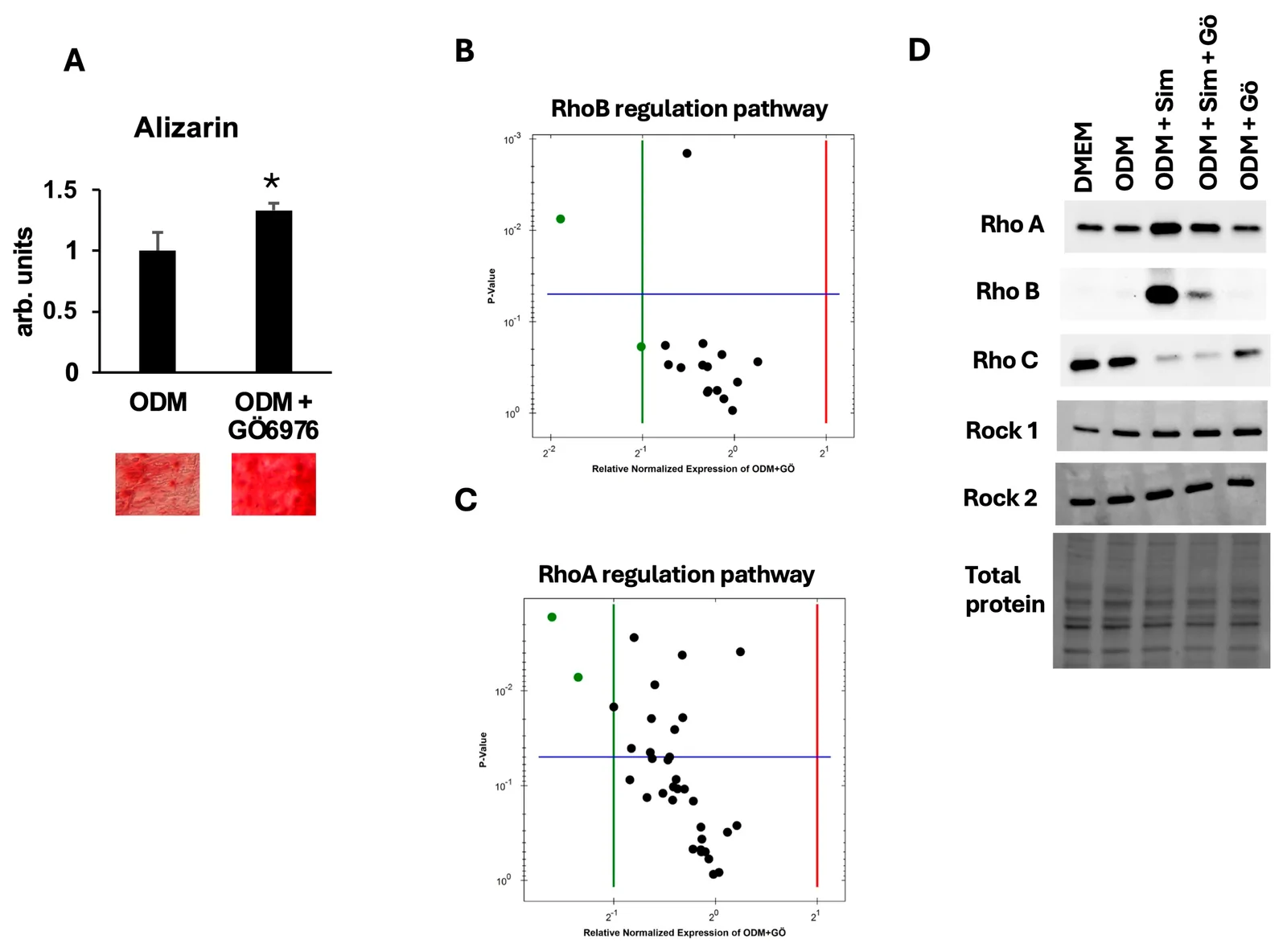
GÖ6976 enhances mineralization through RhoA activation. (**A**) Alizarin Red staining confirming the pro-osteogenic effect of GÖ6976. (**B**,**C**) PrimePCR Array data reflecting modulated RhoGTPase signaling under GÖ6976 influence. (**D**) Western blot analysis of DFCs treated with Simvastatin in the presence or absence of GÖ6976. GÖ6976 effectively counteracts the Simvastatin-induced changes in RhoA and RhoB protein levels. No effects were observed for RhoC, ROCK1, or ROCK2. Differences were considered statistically significant at *p* < 0.05 and marked with an asterisk (*).

Since GÖ6976 is known to enhance DFC mineralization (Figure 5A), we investigated its impact on Rho activation. Treatment with GÖ6976 modulated RhoGTPase signaling at the transcriptional level (Figure 5B,C). To further validate this, we used Simvastatin as a known regulator of RhoGTPase protein expression. While Simvastatin altered RhoA and RhoB expression, these effects were effectively counteracted by GÖ6976 (Figure 5D), whereas RhoC, ROCK1, and ROCK2 remained unaffected.

### 3.4. Inhibitor for RhoGTPase Signaling Counteracts the Pro-Osteogenic Effects of GÖ6976

**Figure 6:**
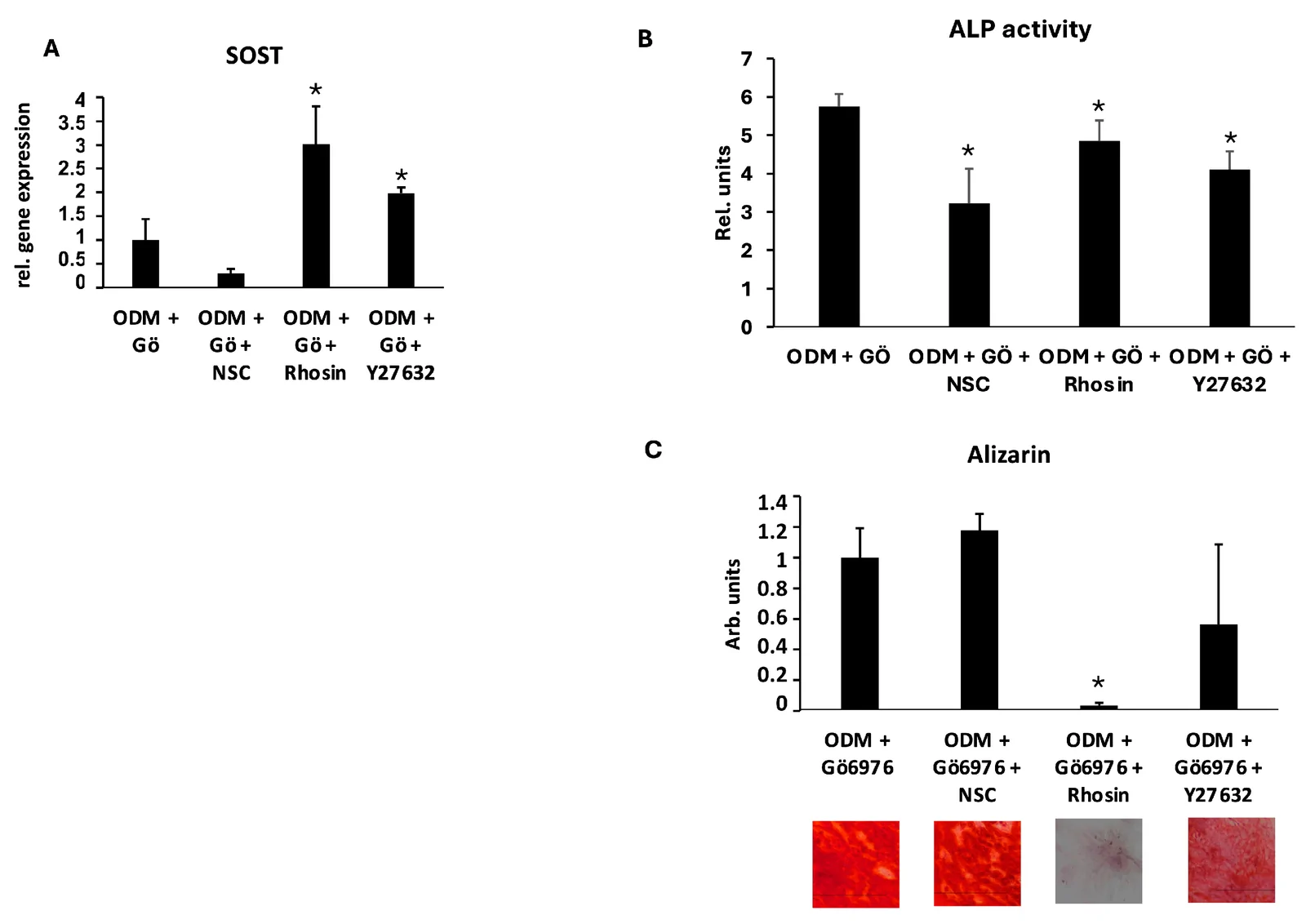
Inhibition of RhoA and Rock antagonize the pro-osteogenic effects of GÖ6976. (**A**) Rhosin and Y27632 induce the expression of *SOST* in GÖ6976-treated cells., an inhibitor of mineralization that is normally suppressed by GÖ6976. (**B**,**C**) Functional characterization using the RhoGTPase-specific inhibitors. All tested RhoGTPase inhibitors decreased the ALP activity (**B**) and Rhosin impaired significantly mineralization (Alizarin Red; (**C**)). These data indicate that RhoA/Rock activation is a prerequisite for the GÖ6976-mediated differentiation boost. Differences were considered statistically significant at *p* < 0.05 and marked with an asterisk (*).

The functional link between GÖ6976 and RhoA was confirmed using the RhoA-specific inhibitor Rhosin. Rhosin significantly inhibited ALP activity and mineralization (Figure 6), thereby mimicking the loss of the osteogenic phenotype. Notably, Rhosin induced SOST expression, which is normally suppressed by GÖ6976 during differentiation. In summary, our results demonstrate that GÖ6976 exerts its positive influence on DFC differentiation primarily through the activation of RhoGTPases, specifically RhoA.

## 4. Discussion

DFCs are utilized as a cellular model to elucidate the complex network of signaling pathways involved in osteogenic differentiation [26,27,28]. In a previous publication, the pro-osteogenic potential of the classical PKC inhibitor GÖ6976 in DFCs was successfully documented [14]. That study revealed that PKC inhibition promotes mineralization; however, a specific mechanism of action could not be established at the time. A more recent study identified the regulation of sclerostin as a potential—or at least partial—mechanism of action. This inhibitor of differentiation was significantly suppressed by GÖ6976, though the downstream signaling pathway remained unidentified at that time [18].

In the present study, utilizing an unbiased phosphoproteomic approach combined with functional assays, we demonstrate for the first time that GÖ6976 promotes DFC mineralization through the targeted, context-dependent activation of the Rho-GTPase signaling pathway and the regulation of sclerostin expression. The phosphoproteomic screening revealed the regulation of proteins associated with the RhoGTPase signaling cascade and cytoskeletal organization. Interestingly, while the total protein expression levels of various RhoGTPases (RhoA, RhoC, Cdc42) remained relatively stable, PrimePCR arrays demonstrated pronounced transcriptional activation specifically within the RhoA regulatory pathway. A central finding of our study is the highly specific, context-dependent correlation of RhoGTPases and osteogenic differentiation after inhibition of PKC with GÖ6976. Once the osteogenic program is initiated, the cellular proteome changes; consequently, Gö6976-mediated PKC inhibition can act as a molecular switch that triggers pro-osteogenic Rho GTPase cascades (including RhoA)—a mechanism demonstrated by the inhibition of ALP activity and Alizarin staining using Rho GTPase inhibitors. However, inhibition of Rho GTPase signaling proteins using NSC23766, Y27632, and Rhosin during differentiation in ODM did not significantly impair either ALP activity or mineralization. Nevertheless, inhibition of the downstream effector ROCK using Y27632 significantly induced the expression of sclerostin (SOST)—a potent inhibitor of the Wnt/β-catenin signaling pathway known to suppress mineralization [18]. Crucially, direct activation of Rho GTPases using GGPP suppressed SOST expression and significantly induced osteogenic differentiation.

Interestingly, a previous study with osteogenic progenitor cells showed similar results. Wang et al. [21] demonstrated that the administration of Y-27632 massively promotes the in vitro expansion and migration of periodontal ligament cells via the ERK cascade, while temporarily pausing their differentiation. Most recently, Zhao et al. [29] showed that *SEPT11*—which is downregulated in patients with osteoporosis—significantly enhances the osteogenic differentiation of mesenchymal stem cells upon artificial overexpression by directly modulating the PI3K/AKT and RhoA/ROCK signaling pathways; furthermore, it has been demonstrated in a mouse model that it improves bone density. The functional significance of ROCK activation—downstream of RhoA—is particularly evident in specialized tissues such as the periodontal ligament. During the osteogenic stimulation a massive reorganization of the cytoskeleton occurs through the induction of F-actin stress fibers [20]. If this process is blocked by the ROCK inhibitor Y-27632, the expression of essential ECM genes—such as Fibronectin-1 and Collagen Type I—drops drastically, thereby almost completely reversing the osteogenic phenotype. Another study showed that in vivo, RhoA-mediated mechanotransduction proves indispensable in response to tensile stimuli, such as those employed in calvarial transsutural distraction osteogenesis for the correction of cranial defects. In a tensile model, Ye et al. demonstrated that mechanical tension stimulates bone formation via the RhoA/ROCK signaling axis [22].

The critical link between GÖ6976 and the RhoGTPase signaling pathway was validated through rescue and stability studies. By utilizing simvastatin to destabilize Rho GTPases via the inhibition of isoprenylation, we observed distinct changes in the expression of RhoA and RhoB. Previous studies have demonstrated that the balance of RhoA activation is sensitive to metabolic and cellular disturbances [30,31]. Remarkably, the co-administration of GÖ6976 completely abrogated these simvastatin-induced changes—particularly for RhoA and RhoB—while RhoC, ROCK1, and ROCK2 remained unaffected. This targeted protective effect suggests that GÖ6976-mediated signaling directly influences the regulation of RhoA and RhoB expression. Our functional assays employing RhoA- and ROCK-specific inhibitors provided clear evidence supporting the involvement of the RhoA/ROCK axis. The inhibitors of RhoA and partly of Rock reduced the GÖ6976-induced increase in ALP activity and mineralization. Mechanistically, GÖ6976 suppresses SOST, at least in part, via the RhoA/ROCK axis.

To investigate how GÖ6976 modulates downstream signaling, we closely evaluated our phosphoproteomic data. We identified significant phosphorylation shifts in a distinct cluster of proteins tightly linked to cytoskeletal restructuring and RhoGTPase regulation, specifically CIP4 (Q15642), Tubulin alpha-1B (P68363), Tubulin beta (Q9BQE3), and GPS1 (Q13098). While the precise biochemical cascade in dental follicle cells remains to be fully elucidated, these data demonstrate that GÖ6976 alters key structural and regulatory components of the RhoA network. This architectural remodeling provides a plausible mechanistic link that aligns with the transcriptional changes seen in our PCR arrays and the downstream suppression of SOST.

In summary, our phosphoproteomics data identified Rho GTPase signaling as a downstream target associated with PKC inhibition. Furthermore, our functional and expression data support a model in which the pro-osteogenic enhancement by GÖ6976 in DFCs is linked to the activation of the RhoA/ROCK axis. Within this osteogenic microenvironment, GÖ6976-induced RhoA/ROCK signaling correlates with the suppression of the mineralization inhibitor SOST, ultimately promoting the accelerated osteogenic differentiation of DFCs.

Finally, several limitations of this study should be acknowledged. First, while phosphoproteomics provides powerful, unbiased insights into signaling cascades, it only captures a temporal and spatial snapshot of the highly complex molecular modifications occurring during DFC differentiation. Second, our mechanistic findings rely primarily on pharmacological interventions and descriptive correlations. It should be noted that GGPP is not strictly RhoA-specific, as it promotes the geranylgeranylation of multiple small GTPases. To definitively map the precise upstream and downstream dynamics of the RhoGTPase network—such as the exact involvement of specific GEFs, GAPs, or the direct transcriptional regulation of the SOST promoter—further genetic validation via loss- and gain-of-function models will be required in future studies.

## Figures and Tables

**Table 1:** Functional Annotation and Pathway Enrichment of the DFC Phosphoproteome (ODM vs. DMEM); Gene Ontology (GO) Biological Processes of Regulated Phosphoproteins.

Term	Count	*p*-Value	Identified Proteins (Accession Numbers)
intracellular protein localization	7	0.000139	Q16181, Q13501, Q96IF1, Q8TEW0, Q86YS7, P35222, Q9UHD8
cell division	8	0.0016	Q16181, Q8TEW0, P27816, Q9NTI5, Q9UHD8, O43491, Q92974, Q32P44
actin cytoskeleton organization	6	0.00255	Q6DN90, Q8IZ21, O75167, Q8TE77, Q9UHD8, Q08AE8
microtubule cytoskeleton organization	5	0.00387	Q7KZI7, Q6VN20, Q8TEW0, P27816, Q32P44
intracellular signal transduction	7	0.0142	Q7KZI7, Q13501, Q9Y2U5, P17096, Q9Y4K4, Q9NQ66, P31323
Wnt signaling pathway	5	0.0144	Q7KZI7, Q96PE1, P35222, O60716, Q96B18

**Table 2:** Functional Annotation and Pathway Enrichment of the DFC Phosphoproteome (ODM vs. DMEM); Reactome Pathway Analysis of Differentially Phosphorylated Sites.

Term	Count	*p*-Value	Identified Proteins (Accession Numbers)
Signal Transduction	25	0.000157	Q13501, Q07065, Q6VN20, Q2M1Z3, Q96B36, Q14643, Q96BY6, P49796, Q8TEW0, Q9Y6M7, Q9NQ66, P35222, Q9Y4F1, O60716, P31323, Q16181, Q99961, Q7L9L4, Q8IYB3, P08123, O14950, O14974, Q9BXK1, Q92974
Signaling by Rho GTPases	10	0.00415	Q07065, Q2M1Z3, Q96BY6, Q8IYB3, Q9Y6M7, Q9Y4F1, O14950, P35222, O14974, Q92974
Signaling by Rho GTPases, Miro GTPases and RHOBTB3	10	0.0048	Q07065, Q2M1Z3, Q96BY6, Q8IYB3, Q9Y6M7, Q9Y4F1, O14950, P35222, O14974, Q92974
RHO GTPase cycle	7	0.0158	Q07065, Q2M1Z3, Q96BY6, Q8IYB3, Q9Y6M7, Q9Y4F1, Q92974

**Table 3:** Bioinformatic Analysis of the Inhibitory Effect of Gö6976 on the DFC Phosphoproteome (ODM + Gö6976 vs. ODM); Gene Ontology (GO) Biological Processes Modulated by Gö6976.

Term	Count	*p*-Value	Identified Proteins (Accession Numbers)
ERAD pathway	3	0.00567	P14625, Q9H0K1, Q9UKM7
microtubule cytoskeleton organization	3	0.0155	P68363, P46821, Q9Y2K2
regulation of translation	3	0.0189	Q9NYF8, Q8NC51, P51116
intracellular signal transduction	4	0.0203	Q9Y6D5, Q13546, Q9H0K1, Q9Y2K2
protein phosphorylation	3	0.0375	Q2M2I8, Q9H0K1, Q9Y2K2

**Table 4:** Bioinformatic Analysis of the Inhibitory Effect of Gö6976 on the DFC Phosphoproteome (ODM + Gö6976 vs. ODM); Reactome Pathway Mapping of Differential Phosphorylation under PKC Inhibition.

Term	Count	*p*-Value	Identified Proteins (Accession Numbers)
Vesicle-mediated transport	6	0.00365	P14625, P68363, Q2M2I8, Q15642, Q13098, P02794
Membrane Trafficking	5	0.0129	P68363, Q2M2I8, Q15642, Q13098, P02794
Post-translational protein modification	7	0.0189	Q15084, O43852, Q13546, P14625, P68363, Q9UKM7, Q13098
RHO GTPase cycle	4	0.0273	Q5JTV8, P68363, Q15642, Q13098
Metabolism of proteins	8	0.0287	Q9Y6D5, Q15084, O43852, Q13546, P14625, P68363, Q9UKM7, Q13098
Cellular responses to stimuli	5	0.0406	P21291, P18206, Q15084, P14625, P68363

## Data Availability

The mass spectrometry proteomics data have been deposited into the ProteomeXchange Consor-tium via the PRIDE partner repository with the dataset identifier PXD081715. The phosphoproteomic datasets generated during the current study are available in the Zenodo repository at zenodo.org (https://doi.org/10.5281/zenodo.21128464).

## Supplementary Materials

The following supporting information can be downloaded at: https://www.mdpi.com/article/10.3390/biomedicines14081769/s1.
