# Supplementary material for: PKC Inhibition by Gö6976 Promotes Osteogenic Differentiation of Dental Follicle Cells Involving Rho-Dependent Pathway Dynamics
Source: Biomedicines. 2026 Aug 6;14(8):1769. doi: 10.3390/biomedicines14081769 (PMC13509162; doi:10.3390/biomedicines14081769)
Supplement: Supplementary file 1 [file biomedicines-14-01769-s001.zip › biomedicines-4374900-supplementary.pdf]

Table S1: regulated Peptides groups ODM vs. DMEM

**User ID**	**Gene Name**
A0FGR8	extended synaptotagmin 2(ESYT2)
O00391	quiescin sulfhydryl oxidase 1(QSOX1)
O00461	golgi integral membrane protein 4(GOLIM4)
O14950	myosin light chain 12B(MYL12B)
O14974	protein phosphatase 1 regulatory subunit 12A(PPP1R12A)
O43491	erythrocyte membrane protein band 4.1 like 2(EPB41L2)
O60238	BCL2 interacting protein 3 like(BNIP3L)
O60336	mitogen-activated protein kinase binding protein 1(MAPKBP1)
O60716	catenin delta 1(CTNND1)
O75167	phosphatase and actin regulator 2(PHACTR2)
O94819	kelch repeat and BTB domain containing 11(KBTBD11)
P08123	collagen type I alpha 2 chain(COL1A2)
P13611	versican(VCAN)
P17096	high mobility group AT-hook 1(HMGA1)
P23193	transcription elongation factor A1(TCEA1)
P27816	microtubule associated protein 4(MAP4)
P27824	calnexin(CANX)
P29966	myristoylated alanine rich protein kinase C substrate(MARCKS)
P31323	protein kinase cAMP-dependent type II regulatory subunit beta(PRKAR2B)
P33527	ATP binding cassette subfamily C member 1 (ABCC1 blood group)(ABCC1)
P35222	catenin beta 1(CTNNB1)
P49796	regulator of G protein signaling 3(RGS3)
P51659	hydroxysteroid 17-beta dehydrogenase 4(HSD17B4)
P53985	solute carrier family 16 member 1(SLC16A1)
Q07065	cytoskeleton associated protein 4(CKAP4)
Q08AE8	spire type actin nucleation factor 1(SPIRE1)
Q09666	AHNAK nucleoprotein(AHNAK)
Q13425	syntrophin beta 2(SNTB2)
Q13501	sequestosome 1(SQSTM1)
Q14195	dihydropyrimidinase like 3(DPYSL3)
Q14498	RNA binding motif protein 39(RBM39)
Q14643	inositol 1,4,5-trisphosphate receptor type 1(ITPR1)
Q15678	protein tyrosine phosphatase non-receptor type 14(PTPN14)
Q16181	septin 7(SEPTIN7)
Q2M1Z3	Rho GTPase activating protein 31(ARHGAP31)
Q32P44	EMAP like 3(EML3)
Q4G0J3	La ribonucleoprotein 7, transcriptional regulator(LARP7)
Q4V9L6	transmembrane protein 119(TMEM119)
Q5SW79	centrosomal protein 170(CEP170)
Q5T4S7	ubiquitin protein ligase E3 component n-recognin 4(UBR4)
Q6DN90	IQ motif and Sec7 domain ArfGEF 1(IQSEC1)
Q6P995	family with sequence similarity 171 member B(FAM171B)
Q6UX15	layilin(LAYN)

Q6VN20	RAN binding protein 10(RANBP10)
Q7KZI7	microtubule affinity regulating kinase 2(MARK2)
Q7L9L4	MOB kinase activator 1B(MOB1B)
Q7L9L4	MOB kinase activator 1A(MOB1A)
Q7Z5L9	interferon regulatory factor 2 binding protein 2(IRF2BP2)
Q86YS7	C2 calcium dependent domain containing 5(C2CD5)
Q8IU81	interferon regulatory factor 2 binding protein 1(IRF2BP1)
Q8IUD2	ELKS/RAB6-interacting/CAST family member 1(ERC1)
Q8IYB3	serine and arginine repetitive matrix 1(SRRM1)
Q8IZ21	phosphatase and actin regulator 4(PHACTR4)
Q8TE77	slingshot protein phosphatase 3(SSH3)
Q8TEW0	par-3 family cell polarity regulator(PARD3)
Q92890	ubiquitin recognition factor in ER associated degradation 1(UFD1)
Q92974	Rho/Rac guanine nucleotide exchange factor 2(ARHGEF2)
Q96B18	dishevelled binding antagonist of beta catenin 3(DACT3)
Q96B36	AKT1 substrate 1(AKT1S1)
Q96BY6	dedicator of cytokinesis 10(DOCK10)
Q96BY7	autophagy related 2B(ATG2B)
Q96IF1	ajuba LIM protein(AJUBA)
Q96JP5	ZFP91 zinc finger protein, atypical E3 ubiquitin ligase(ZFP91)
Q96PE1	adhesion G protein-coupled receptor A2(ADGRA2)
Q99961	SH3 domain containing GRB2 like 1, endophilin A2(SH3GL1)
Q9BU76	chromosome 1 open reading frame 35(C1orf35)
Q9BXK1	KLF transcription factor 16(KLF16)
Q9BZL4	protein phosphatase 1 regulatory subunit 12C(PPP1R12C)
Q9BZQ8	niban apoptosis regulator 1(NIBAN1)
Q9H1B7	interferon regulatory factor 2 binding protein like(IRF2BPL)
Q9H4G0	erythrocyte membrane protein band 4.1 like 1(EPB41L1)
Q9H694	BicC family RNA binding protein 1(BICC1)
Q9NQ66	phospholipase C beta 1(PLCB1)
Q9NTI5	PDS5 cohesin associated factor B(PDS5B)
Q9NUQ6	spermatogenesis associated serine rich 2 like(SPATS2L)
Q9P246	stromal interaction molecule 2(STIM2)
Q9UHD8	septin 9(SEPTIN9)
Q9UJU6	drebrin like(DBNL)
Q9UPQ0	LIM and calponin homology domains 1(LIMCH1)
Q9Y282	ERGIC and golgi 3(ERGIC3)
Q9Y2U5	mitogen-activated protein kinase kinase kinase 2(MAP3K2)
Q9Y385	ubiquitin conjugating enzyme E2 J1(UBE2J1)
Q9Y4F1	FERM, ARH/RhoGEF and pleckstrin domain protein 1(FARP1)
Q9Y4F5	centrosomal protein 170B(CEP170B)
Q9Y4K4	mitogen-activated protein kinase kinase kinase kinase 5(MAP4K5)
Q9Y520	proline rich coiled-coil 2C(PRRC2C)
Q9Y6M7	solute carrier family 4 member 7(SLC4A7)
Q9Y6R1	solute carrier family 4 member 4(SLC4A4)

Table S2: Summary of Modifications and Sequence Annotations, ODM vs. DMEM

PROTEIN ACCESSIONS	MODIFICATIONS	POSITION	ANNOTATED SEQUENCE
**P31323**	1xTMTpro [N-Term]; 1xPhospho [S3]; 1xCarbamidomethyl [C5]	[112-132]	[R].RASVCAEAYNPDEEEDDAESR.[I]
**P08123**	1xTMTpro [N-Term]; 1xPhospho [S5]; 1xTMTpro [K14]	[451-464]	[R].GLPGSPGNIGPAGK.[E]
**P27816**	1xTMTpro [N-Term]; 1xDeamidated [N4]; 1xPhospho [S/T]; 1xTMTpro [K11]	[923-933]	[R].LATNTSAPDLK.[N]
**P17096**	2xTMTpro [K1; K4]; 1xTMTpro [N-Term]; 1xPhospho [S15]	[89-107]	[K].KLEKEEEEGISQESSEEEQ.[-]
**Q9BZQ8**	1xTMTpro [N-Term]; 1xPhospho [S7]; 1xTMTpro [K10]	[627-636]	[K].QPEVPSSLAK.[G]
**Q8IZ21**	1xTMTpro [N-Term]; 1xPhospho [S3]	[588-601]	[R].RLSQRPTPEELEQR.[N]
**Q9NUQ6**	1xTMTpro [N-Term]; 1xPhospho [S/T]	[512-524]	[R].QHAADTSEARPFR.[G]
**Q9Y6R1**	1xTMTpro [N-Term]; 1xPhospho [S3]	[1067-1075]	[R].ERSPTFLER.[H]
**A0FGR8**	1xTMTpro [N-Term]; 1xPhospho [S]; 1xTMTpro [K15]	[736-750]	[R].SSSSLLASPGHISVK.[E]
**P51659**	1xTMTpro [N-Term]; 1xPhospho [S3]; 1xTMTpro [K16]	[316-331]	[R].ATSTATSGFAGAIGQK.[L]
**Q92974**	1xTMTpro [N-Term]; 1xPhospho [S2]	[955-962]	[R].LSPPHSPR.[D]
**Q32P44**	1xTMTpro [N-Term]; 1xPhospho [S3]	[174-183]	[K].AISSANLLVR.[S]
**Q09666**	1xTMTpro [N-Term]; 2xPhospho [S/T]; 1xTMTpro [K15]	[5726-5740]	[K].GGVTGSPEASISGSK.[G]
**O43491**	1xTMTpro [N-Term]; 1xPhospho [S]	[46-60]	[K].GSQPPPAAESQSSLR.[R]
**O60716**	1xTMTpro [N-Term]; 1xPhospho [S3]	[250-264]	[R].APSRQDVYGPQPQVR.[V]
**Q9NQ66**	1xTMTpro [N-Term]; 1xPhospho [S3]; 1xTMTpro [K14]	[1197-1210]	[K].TPSSEELGGDIPGK.[E]

**O94819**	1xTMTpro [N-Term]; 1xCarbamidomethyl [C2]; 1xPhospho [S10]	[98-111]	[R].ACPEEPAAPSPEPR.[V]
**Q2M1Z3**	1xPhospho [S1]; 1xTMTpro [N-Term]	[1432-1438]	[R].SVILDGR.[S]
**Q9Y282**	1xTMTpro [N-Term]; 1xPhospho [S6]	[111-121]	[K].DGIPVSSEAER.[H]
**P31323**	1xTMTpro [N-Term]; 1xPhospho [S3]; 1xCarbamidomethyl [C5]; 1xDeamidated [N10]	[112-132]	[R].RASVCAEAYNPDEEEDDAESR.[I]
**Q86YS7**	1xTMTpro [N-Term]; 1xCarbamidomethyl [C11]; 1xPhospho [S13]; 1xTMTpro [K16]	[248-263]	[K].LSSPAAFLPACNSPSK.[E]
**Q9P246**	1xTMTpro [N-Term]; 1xPhospho [S]	[518-528]	[R].SIVPSSPQPQR.[A]
**O60716**	1xTMTpro [N-Term]; 1xPhospho [S15]	[216-233]	[R].HYEDGYPGGSDNYGSLSR.[V]
**P23193**	1xTMTpro [K1]; 1xTMTpro [N-Term]; 1xPhospho [S10]	[91-104]	[K].KEPAITSQNSPEAR.[E]
**O14974**	1xTMTpro [N-Term]; 1xPhospho [T2]; 1xTMTpro [K15]	[695-709]	[R].STQGVTLTDLQEAEK.[T]
**P53985**	1xTMTpro [N-Term]; 1xPhospho [S5]; 2xTMTpro [K11; K17]	[463-479]	[K].EEETSIDVAGKPNEVTK.[A]
**Q9BU76**	1xTMTpro [N-Term]; 1xPhospho [S/T]	[210-225]	[R].RPAEATSSPTSPERPR.[H]
**P27824**	1xTMTpro [N-Term]; 1xDeamidated [N8]; 1xPhospho [S10]	[574-585]	[K].AEEDEILNRSPR.[N]
**Q9Y520**	1xTMTpro [N-Term]; 1xPhospho [S]	[1542-1551]	[R].SFSSQRPVDR.[Q]
**P29966**	1xTMTpro [N-Term]; 1xPhospho [S/T]; 3xTMTpro [K15; K16; K17]	[138-154]	[K].AEDGATPSPSNETPKKK.[K]
**O60716**	1xTMTpro [N-Term]; 1xPhospho [S]	[265-274]	[R].VGGSSVDLHR.[F]
**Q5T4S7**	1xTMTpro [N-Term]; 2xPhospho [T3; S7]	[2713-2721]	[R].HVTLPSSPR.[S]
**Q8IU81**	1xPhospho [S1]; 1xTMTpro [N-Term]; 1xTMTpro [K8]	[66-73]	[R].SPGPPALK.[H]
**Q9Y4K4**	1xTMTpro [N-Term]; 1xPhospho [S/T]; 1xTMTpro [K9]	[333-341]	[R].TASEINFDK.[L]

**Q6UX15**	1xTMTpro [N-Term]; 1xPhospho [S2]; 1xTMTpro [K14]	[298-311]	[K].QSEADLAETRPDLK.[N]
**Q14643**	1xTMTpro [N-Term]; 1xPhospho [S3]	[1596-1604]	[R].RDSVLAASR.[D]
**Q9UHD8**	1xTMTpro [N-Term]; 1xPhospho [T4]	[46-53]	[R].RVQTPLLR.[A]
**Q96B18**	1xTMTpro [N-Term]; 1xPhospho [S7]	[499-511]	[R].VPGPGPSPSAPQR.[R]
**Q08AE8**	1xTMTpro [N-Term]; 1xPhospho [T7]	[503-514]	[K].FLPISSTPQPER.[R]
**O43491**	1xPhospho [S/T/Y]; 1xTMTpro [N-Term]; 1xTMTpro [K8]	[87-94]	[K].SYTLVVAK.[D]
**Q13501**	1xTMTpro [N-Term]; 2xPhospho [S/T]; 1xTMTpro [K14]	[268-281]	[R].LTPVSPESSSTEEK.[S]
**Q13425**	1xTMTpro [N-Term]; 1xPhospho [S9]	[102-114]	[R].GPAGEAGASPPVR.[R]
**O43491**	1xTMTpro [N-Term]; 1xPhospho [S]; 1xDeamidated [Q11]	[46-60]	[K].GSQPPPAAESQSSLR.[R]
**Q8IYB3**	1xTMTpro [N-Term]; 2xPhospho [S3; S5]	[387-396]	[R].RLSPSASPPR.[R]
**O00391**	1xTMTpro [N-Term]; 1xPhospho [S6]; 1xTMTpro [K11]	[421-431]	[R].QNVDHSQEAAK.[A]
**Q9BZL4**	1xTMTpro [N-Term]; 1xPhospho [S/T]; 1xTMTpro [K11]	[559-569]	[R].STQGVTLTDLK.[E]
**Q9BXK1**	1xTMTpro [N-Term]; 1xPhospho [S]	[89-114]	[R].GGPGAAPGGASPASSSSAASSPSSGR.[A]
**Q6VN20**	1xTMTpro [N-Term]; 1xPhospho [S8]	[358-371]	[K].SQDSYPGSPSLSPR.[H]
**Q9H1B7**	2xTMTpro [K1; K15]; 1xTMTpro [N-Term]; 1xPhospho [S3]	[545-559]	[R].KASPEPPDSAEGALK.[L]
**P23193**	1xTMTpro [N-Term]; 1xPhospho [S9]	[92-104]	[K].EPAITSQNSPEAR.[E]
**Q9BZL4**	1xTMTpro [N-Term]; 1xPhospho [S/T]; 1xTMTpro [K12]	[558-569]	[R].RSTQGVTLTDLK.[E]
**P27824**	1xTMTpro [N-Term]; 1xPhospho [S10]	[574-585]	[K].AEEDEILNRSPR.[N]
**Q9NTI5**	1xTMTpro [N-Term]; 1xPhospho [S3]; 1xTMTpro [K18]	[1356-1373]	[R].AESPESSAIESTQSTPQK.[G]

**Q96B36**	1xTMTpro [N-Term]; 1xPhospho [T3]; 1xTMTpro [K8]	[244-251]	[R].LNTSDFQK.[L]
**P49796**	1xTMTpro [N-Term]; 1xPhospho [S3]	[941-952]	[R].THSEGSLLQEPR.[G]
**Q4V9L6**	1xTMTpro [N-Term]; 1xPhospho [S]	[157-170]	[R].APDSRPEEALDSSR.[Q]
**O60238**	1xTMTpro [N-Term]; 1xPhospho [S]; 1xTMTpro [K15]	[115-129]	[R].DHSSQSEEEVVEGEK.[E]
**P23193**	1xTMTpro [K1]; 1xTMTpro [N-Term]; 1xDeamidated [N9]; 1xPhospho [S10]	[91-104]	[K].KEPAITSQNSPEAR.[E]
**Q8IYB3**	1xTMTpro [N-Term]; 1xPhospho [S/Y]	[595-602]	[R].RYSPPIQR.[R]
**Q96PE1**	1xTMTpro [N-Term]; 1xPhospho [S12]	[965-978]	[R].ASLEAGEELRGSTR.[L]
**Q15678**	1xTMTpro [N-Term]; 1xPhospho [S]	[455-467]	[R].GILHTDSQSQSLR.[N]
**P49796**	1xTMTpro [N-Term]; 1xPhospho [S4]	[940-952]	[R].RTHSEGSLLQEPR.[G]
**Q9Y4F1**	1xTMTpro [N-Term]; 1xPhospho [T11]	[14-27]	[R].LGAPENSGISTLER.[G]
**Q8IUD2**	1xTMTpro [N-Term]; 1xDeamidated [Q6]; 1xPhospho [S11]	[11-23]	[K].VEPSSQSPGRSPR.[L]
**Q8TE77**	1xTMTpro [N-Term]; 1xPhospho [S3]	[35-42]	[R].RQSFAVLR.[G]
**Q7Z5L9**	1xTMTpro [N-Term]; 1xPhospho [S4]	[237-252]	[K].RPASVSSSAAVEHEQR.[E]
**P33527**	1xTMTpro [N-Term]; 1xPhospho [S]	[913-926]	[R].QLSSSSSYSGDISR.[H]
**Q6DN90**	1xTMTpro [N-Term]; 1xPhospho [S/T]	[84-92]	[K].LQHSTSILR.[K]
**Q96JP5**	1xTMTpro [N-Term]; 1xPhospho [S]; 1xTMTpro [K20]	[81-100]	[R].RSSPSARPPDVPGQQPQAAK.[S]
**Q5SW79**	1xTMTpro [N-Term]; 1xPhospho [S/T]; 1xTMTpro [K13]	[1055-1067]	[R].TPLTSADEHVHSK.[L]
**Q9Y4F1**	1xTMTpro [N-Term]; 1xPhospho [S12]	[878-897]	[K].SPDEATAADQESEDDLSASR.[T]
**Q8IYB3**	1xTMTpro [N-Term]; 1xPhospho [S/T]	[690-701]	[R].APQTSSSPPPVR.[R]
**Q92974**	1xTMTpro [N-Term]; 1xPhospho [S5]	[928-936]	[R].QELGSPEER.[L]

**Q6P995**	1xTMTpro [N-Term]; 1xPhospho [S/T]; 1xTMTpro [K17]	[400-416]	[K].RDQTTSTTHINHISTVK.[V]
**O14974**	1xTMTpro [N-Term]; 1xPhospho [S/T]; 1xDeamidated [Q]; 1xTMTpro [K16]	[694-709]	[R].RSTQGVTLTDLQEAEK.[T]
**Q7KZI7**	1xTMTpro [N-Term]; 1xPhospho [S3]; 1xTMTpro [K9]	[629-637]	[R].GASGSIFSK.[F]
**O14974**	1xTMTpro [N-Term]; 1xPhospho [S/T]; 1xTMTpro [K16]	[694-709]	[R].RSTQGVTLTDLQEAEK.[T]
**Q9UPQ0**	1xPhospho [S1]; 1xTMTpro [N-Term]; 1xTMTpro [K8]	[377-384]	[R].SVSQDLIK.[K]
**O60336**	1xTMTpro [N-Term]; 1xPhospho [S3]	[1196-1207]	[K].AQSVHSLVPQER.[H]
**Q14498**	1xTMTpro [N-Term]; 1xPhospho [S3]; 1xTMTpro [K9]	[95-103]	[R].YRSPYSGPK.[F]
**O43491**	1xTMTpro [N-Term]; 1xPhospho [S]	[46-61]	[K].GSQPPPAAESQSSLRR.[Q]
**O14974**	1xTMTpro [N-Term]; 1xPhospho [T3]; 2xDeamidated [Q4; Q12]; 1xTMTpro [K16]	[694-709]	[R].RSTQGVTLTDLQEAEK.[T]
**Q96IF1**	1xTMTpro [N-Term]; 1xPhospho [S8]; 1xCarbamidomethyl [C15]	[256-275]	[R].GAQPGRHSVTGYGDCAVGAR.[Y]
**Q9UJU6**	1xTMTpro [N-Term]; 1xPhospho [T3]	[289-299]	[K].QLTQPETHFGR.[E]
**O60716**	1xTMTpro [N-Term]; 1xPhospho [S8]; 1xTMTpro [K11]	[345-355]	[R].GSLASLDSLRK.[G]
**Q07065**	1xTMTpro [N-Term]; 1xPhospho [S/T]	[227-240]	[R].ERDFTSLENTVEER.[L]
**Q4G0J3**	1xTMTpro [N-Term]; 1xPhospho [S8]; 1xTMTpro [K17]	[254-270]	[R].SRPTSEGSDIESTEPQK.[Q]
**P27824**	1xTMTpro [N-Term]; 1xPhospho [S10]; 1xDeamidated [N13]	[574-587]	[K].AEEDEILNRSPRNR.[K]
**Q9Y4F5**	1xPhospho [S1]; 1xTMTpro [N-Term]	[772-781]	[R].SPQEGPTWSR.[G]
**Q9Y4F1**	1xTMTpro [N-Term]; 1xPhospho [S11]	[417-431]	[K].VSAGEPGSHPSPAPR.[R]

**Q16181**	1xTMTpro [N-Term]; 1xDeamidated [N/Q]; 1xPhospho [T10]; 1xTMTpro [K13]	[417-429]	[R].ILEQQNSSRTLEK.[N]
**Q9Y6M7**	1xTMTpro [N-Term]; 1xPhospho [S6]	[79-93]	[K].EDGRESPSYDTPSQR.[V]
**O43491**	1xTMTpro [N-Term]; 2xDeamidated [N5; N8]; 1xPhospho [S]; 1xTMTpro [K17]	[29-45]	[K].EVAENQQNQSSDPEEEK.[G]
**O00461**	1xTMTpro [N-Term]; 2xTMTpro [K12; K17]; 1xPhospho [S14]	[570-586]	[R].EENLPDENEEQKQSNQK.[Q]
**P33527**	1xTMTpro [N-Term]; 1xPhospho [S4]; 1xTMTpro [K10]	[927-936]	[R].HHNSTAELQK.[A]
**P35222**	1xTMTpro [N-Term]; 1xPhospho [S3]; 1xOxidation [M4]	[550-565]	[R].RTSMGGTQQQFVEGVR.[M]
**Q7L9L4**	1xTMTpro [N-Term]; 1xPhospho [T5]	[31-42]	[K].HAEATLGSGNLR.[MQ]
**Q9H694**	1xTMTpro [N-Term]; 1xPhospho [S10]	[962-973]	[R].QYHSDIASVSGR.[W]
**O75167**	1xTMTpro [N-Term]; 1xPhospho [S3]	[520-533]	[R].RLSQRPTTEELEQR.[N]
**Q8IYB3**	1xTMTpro [N-Term]; 2xPhospho [S4; T8]; 1xTMTpro [K12]	[399-410]	[R].HRPSPPATPPPK.[T]
**O14950**	1xTMTpro [N-Term]; 1xPhospho [S3]; 1xTMTpro [K18]	[18-35]	[R].ATSNVFAMFDQSQIQEFK.[E]
**Q9Y2U5**	1xTMTpro [N-Term]; 1xPhospho [S3]	[151-160]	[K].RLSIIGPTSR.[D]
**Q96BY6**	1xPhospho [S1]; 1xTMTpro [N-Term]	[12-23]	[R].SLLRPGQAAELR.[H]
**P13611**	1xOxidation [M1]; 1xTMTpro [N-Term]; 1xPhospho [S15]; 1xTMTpro [K16]	[1337-1352]	[R].MSDLSVIGHPIDSESK.[E]
**Q09666**	1xTMTpro [N-Term]; 1xPhospho [S/T]; 1xTMTpro [K10]	[5822-5831]	[K].FGTFGGLGSK.[S]
**Q9Y385**	1xTMTpro [N-Term]; 1xPhospho [S/T]	[264-278]	[R].RLSTSPDVIQGHQPR.[D]
**Q14195**	1xTMTpro [N-Term]; 1xPhospho [S2]; 1xDeamidated [N7]	[521-531]	[R].GSPTRPNPPVR.[N]

**Q92890**	1xTMTpro [N-Term]; 1xPhospho [S10]	[290-301]	[R].FVAFSGEGQSLR.[K]
**Q99961**	1xTMTpro [N-Term]; 1xPhospho [S]	[283-290]	[K].IAASSSFR.[S]
**O14950**	1xTMTpro [N-Term]; 1xPhospho [S/T]; 1xOxidation [M8]; 1xTMTpro [K18]	[18-35]	[R].ATSNVFAMFDQSQIQEFK.[E]
**Q8TEW0**	1xTMTpro [N-Term]; 1xPhospho [Y4]	[196-204]	[K].DENYRSLPR.[D]
**Q9H4G0**	1xTMTpro [N-Term]; 1xPhospho [S4]; 1xTMTpro [K9]	[507-515]	[K].HQASINELK.[R]
**Q7Z5L9**	1xTMTpro [N-Term]; 2xTMTpro [K2; K15]; 1xPhospho [S4]	[357-371]	[K].RKPSPEPEGEVGPPK.[I]
**Q96BY7**	1xTMTpro [N-Term]; 1xPhospho [S2]; 1xCarbamidomethyl [C5]; 1xTMTpro [K15]	[239-253]	[K].SSPVCSTAPVETEPK.[L]

Table S3: regulated Peptides groups ODM vs. ODM + GÖ

**User ID**	**Gene Name**
O43852	calumenin(CALU)
P02794	ferritin heavy chain 1(FTH1)
P14625	heat shock protein 90 beta family member 1(HSP90B1)
P18206	vinculin(VCL)
P21291	cysteine and glycine rich protein 1(CSRP1)
P46821	microtubule associated protein 1B(MAP1B)
P51116	FMR1 autosomal homolog 2(FXR2)
P68363	tubulin alpha 1b(TUBA1B)
Q0ZGT2,Q0ZGT2	nexilin F-actin binding protein(NEXN)
Q13098	G protein pathway suppressor 1(GPS1)
Q13546	receptor interacting serine/threonine kinase 1(RIPK1)
Q15084	protein disulfide isomerase family A member 6(PDIA6)
Q15642	thyroid hormone receptor interactor 10(TRIP10)
Q2M2I8,Q2M2I8	AP2 associated kinase 1(AAK1)
Q5JTV8	torsin 1A interacting protein 1(TOR1AIP1)
Q8NC51	SERPINE1 mRNA binding protein 1(SERBP1)
Q8TEQ0	sorting nexin 29(SNX29)
Q8WUF5	protein phosphatase 1 regulatory subunit 13 like(PPP1R13L)
Q9BXP5	serrate, RNA effector molecule(SRRT)
Q9H0K1	salt inducible kinase 2(SIK2)
Q9NYF8	BCL2 associated transcription factor 1(BCLAF1)
Q9NZM1	myoferlin(MYOF)
Q9UKM7	mannosidase alpha class 1B member 1(MAN1B1)
Q9UQ35	serine/arginine repetitive matrix 2(SRRM2)
Q9Y2K2	SIK family kinase 3(SIK3)
Q9Y6D5	ARF guanine nucleotide exchange factor 2(ARFGEF2)

Table S4 Summary of Modifications and Sequence Annotations; ODM + GÖ vs ODM

PROTEIN ACCESSION	MODIFICATIONS	POSITION	ANNOTATED SEQUENCE
**Q9Y6D5**	1xTMTpro [N-Term]; 1xPhospho [S]	[345-365]	[R].QSLSSADNLESDAQGHQVAAR.[F]
**O43852**	1xTMTpro [N-Term]; 1xDeamidated [N/Q]; 2xPhospho [S7; Y10]; 1xTMTpro [K22]	[38-59]	[K].VHNDAQSFDYDHDAFLGAEEAK.[T]
**O43852**	1xTMTpro [N-Term]; 2xPhospho [S7; Y10]; 1xTMTpro [K22]	[38-59]	[K].VHNDAQSFDYDHDAFLGAEEAK.[T]
**O43852**	1xTMTpro [N-Term]; 1xPhospho [S6]; 1xTMTpro [K10]	[272-281]	[R].HLVYESDQNK.[D]
**P02794**	1xTMTpro [N-Term]; 1xPhospho [S]	[174-183]	[K].HTLGDSDNES.[-]
**P02794**	1xOxidation [M1]; 1xTMTpro [N-Term]; 1xTMTpro [K15]; 1xPhospho [S/T/Y]	[159-183]	[K].MGAPESGLAEYLFDKHTLGDSDNES.[-]
**P14625**	1xTMTpro [N-Term]; 2xTMTpro [K3; K19]; 1xPhospho [S6]	[301-319]	[K].EEKEESDDEAAVEEEEEEK.[K]
**P14625**	1xTMTpro [N-Term]; 1xPhospho [S3]; 1xTMTpro [K16]	[304-319]	[K].EESDDEAAVEEEEEEK.[K]
**P18206**	1xTMTpro [N-Term]; 1xPhospho [S3]; 2xOxidation [M5; M7]; 1xTMTpro [K10]	[793-802]	[K].TISPMVMDAK.[A]
**P21291**	1xTMTpro [N-Term]; 1xPhospho [S12]; 1xTMTpro [K15]	[70-84]	[K].GYGYGQGAGTLSTDK.[G]
**P46821**	1xTMTpro [N-Term]; 1xPhospho [S6]	[990-999]	[K].RESVASGDDR.[A]
**P51116**	1xTMTpro [N-Term]; 1xPhospho [S16]; 1xTMTpro [K22]	[438-459]	[R].TGGPAYGPSSDVSTASETESEK.[R]
**P68363; Q9BQE3**	1xTMTpro [N-Term]; 1xPhospho [S13]; 1xTMTpro [K16]	[265-280]	[R].IHFPLATYAPVISAEK.[A]
**Q0ZGT2**	1xTMTpro [N-Term]; 1xPhospho [S3]; 1xTMTpro [K11]	[363-373]	[K].TISQEFLTPGK.[L]
**Q13098**	1xTMTpro [N-Term]; 1xPhospho [S7]	[468-486]	[K].SPPREGSQGELTPANSQSR.[M]

**Q13546**	1xTMTpro [N-Term]; 1xPhospho [S3]; 1xTMTpro [K22]	[414-435]	[R].RVSHDPFAQQRPYENFQNTEGK.[G]
**Q15084**	1xTMTpro [N-Term]; 1xPhospho [S13]; 1xTMTpro [K22]	[416-437]	[R].DGELPVEDDIDLSDVELDDLGK.[D]
**Q15642**	1xTMTpro [N-Term]; 1xTMTpro [K2]; 1xDeamidated [N21]; 1xPhospho [S25]	[327-353]	[K].NKPRPPPLSPLGGPVPSALPNGPPSPR.[S]
**Q2M2I8**	1xTMTpro [N-Term]; 1xPhospho [S]	[9-37]	[R].REQGGSGLGSGSSGGGGSTSGLGSGYIGR.[V]
**Q2M2I8**	1xTMTpro [N-Term]; 1xPhospho [S]	[10-37]	[R].EQGGSGLGSGSSGGGGSTSGLGSGYIGR.[V]
**Q2M2I8**	1xTMTpro [N-Term]; 1xDeamidated [Q2]; 1xPhospho [S]	[10-37]	[R].EQGGSGLGSGSSGGGGSTSGLGSGYIGR.[V]
**Q5JTV8**	1xTMTpro [N-Term]; 1xPhospho [S14]; 1xOxidation [M17]	[130-148]	[R].LQQQHSEQPPLQPSPVMTR.[R]
**Q8NC51**	1xTMTpro [N-Term]; 1xTMTpro [K2]; 1xPhospho [S]; 1xOxidation [M13]	[328-345]	[K].SKSEEAHAEDSVMDHHFR.[K]
**Q8TEQ0**	1xTMTpro [N-Term]; 1xPhospho [S6]	[805-813]	[R].NVEPQSGDL.[-]
**Q8WUF5**	1xTMTpro [N-Term]; 1xPhospho [S3]; 1xTMTpro [K12]	[290-301]	[R].ESSLDGLGGTGK.[D]
**Q9BXP5**	1xTMTpro [N-Term]; 2xPhospho [S4; S11]; 1xTMTpro [K15]	[64-78]	[R].ERFSPPRHELSPPQK.[R]
**Q9H0K1**	1xTMTpro [N-Term]; 1xPhospho [S3]; 1xTMTpro [K12]	[356-367]	[R].RPSTIAEQTVAK.[A]
**Q9NYF8**	1xTMTpro [N-Term]; 1xPhospho [S/Y]; 1xTMTpro [K11]	[507-517]	[K].DLFDYSPPLHK.[N]
**Q9NZM1**	1xTMTpro [N-Term]; 1xPhospho [S7]	[168-180]	[K].GPVGTVSEAQLAR.[R]
**Q9UKM7**	1xTMTpro [N-Term]; 1xPhospho [S11]; 1xTMTpro [K15]	[164-178]	[R].GPPHLQIRPPSQDLK.[D]
**Q9UQ35**	1xTMTpro [N-Term]; 1xPhospho [S21]; 1xTMTpro [K27]	[303-329]	[R].RGEGDAPFSEPGTTSTQRPSSPETATK.[Q]
**Q9Y2K2**	1xTMTpro [N-Term]; 1xPhospho [S3]; 1xTMTpro [K13]	[624-636]	[R].RFSDGAASIQAFK.[A]
